# Ophthalmic Complications in Maxillofacial Trauma: A Prospective Study

**DOI:** 10.7759/cureus.27608

**Published:** 2022-08-02

**Authors:** Swapnil M Jain, Neelima Gehlot, Arunkumar KV, Pawan Prasad, Prashansa Mehta, Thota Roger Paul, Ankit Dupare, Chakka Satyadev CVNS, Sadaf Rahman

**Affiliations:** 1 Department of Oral and Maxillofacial Surgery, Teerthanker Mahaveer Dental College and Research Centre, Moradabad, IND; 2 Department of Oral and Maxillofacial Surgery, Konaseema Institute of Medical Sciences (KIMS) Dental College and Hospital, Amalapuram, IND; 3 Department of Oral and Maxillofacial Surgery, Rungta College of Dental Sciences and Research, Bhilai, IND; 4 Department of Dentistry, Leaside Orthodontic Centre, Toronto, CAN; 5 Department of Oral and Maxillofacial Surgery, Smile Dental Clinic, New Delhi, IND

**Keywords:** panfacial trauma, ophthalmoplegia, ophthalmic complications, ocular injuries, maxillofacial trauma, blindness

## Abstract

Objectives: To determine the incidence and types of ophthalmic complications associated with maxillofacial trauma over a period of 24 months.

Methods: An institutional prospective study was conducted on 62 patients presenting with maxillofacial trauma to study the correlation between facial trauma and ophthalmic complications.

Results: Road traffic accidents were reported to be the primary etiologic factor for most trauma cases studied. Zygomaticomaxillary complex (ZMC) fracture was associated with more ophthalmic complications while fractures involving the orbital rims and walls were associated with severe complications.

Conclusions: Maxillofacial trauma, particularly those associated with midface, including ZMC fracture, Le Fort II, Le Fort III, and naso-orbito-ethmoidal fractures, can commonly cause ophthalmic complications and blindness in rare cases. Hence, every patient with maxillofacial trauma should undergo an ophthalmic examination and should be placed under close observation for necessary treatment when required.

## Introduction

Globally, one-third of the annual trauma cases include maxillofacial skeletal fractures [1] with 200-300 per 100,000 persons getting hospitalized due to head injury and about 25% of them are accompanied by ocular and visual defects [2]. The human eye, responsible for vision, occupies only 0.3% of the total body surface. Indeed, in the event of a facial injury, particularly to the midface, the eye and its adnexa are defenseless against harm, despite the fat and bone surrounding it. According to Le Fort, the face is resistant to external forces because its tissues are flexible, the fundamental periosteum is present, and the surrounding sensitive tissues provide a protective barrier [3]. Three factors, including the prominent hard periorbita, the globe's extreme design, the optic nerve's strong bone security as it enters the orbit, and the patient's self-preservation, work together to protect the globe from injury, making vision insufficiency after face traumas unusual. There is a broad range of potential triggers and consequences for eye damage in the case of facial cracks [4].

Mandible being the most common, zygomaticomaxillary complex (ZMC) fractures are the second most common kind of facial fractures in maxillofacial injury followed by orbital, naso-orbito-ethmoid (NOE), and Le Fort fractures. Midface injuries such as ZMC fracture, orbital, Le Fort II, and Le Fort III fractures are associated with an increased risk of vision impairment by as much as 6.7 times compared to other breaks in facial bone units [5]. The incidence of visual sequelae in patients with midface cracks ranged from 2.7% to 9.6%. Literature studies have shown a close association between orbito-zygomatic fractures and ocular complications. The midface injuries pose a high risk for visual acuity loss and may cause eye complications resulting in considerable morbidity [6-8]. Management of ocular injuries resulting from midface trauma involves a multidisciplinary approach for outlining a definitive treatment.

Etiology for maxillofacial trauma enlists road traffic accidents (RTAs), assaults, falls, sports injuries, and others [4,8,9]. Industrial accidents have also been reported in a study conducted by Amrith et al. [10]. RTA is a major cause of trauma in developing nations like India. On the contrary, many non-Indian studies confirm assaults as the major etiology for maxillofacial trauma [11]. Male gender predilection for trauma is reported to be significantly more than the female population, accounting for 85% of the cases globally [3,12].

Traumatic optic neuropathy and/or a perforated globe are two extreme cases of ophthalmic problems following trauma. Of patients with midfacial fractures, 20% had significant eye impairment, according to literature reviews [3]. Due to insufficient treatment, the global prevalence of blindness after craniofacial trauma ranges from 0% to 11%, with serious social and medicolegal consequences [13,14]. Every patient with a mid-facial injury should have an ocular exam, and those with suspicious injuries should be brought to an ophthalmologist.

Though ophthalmic injuries are treated by ophthalmologists, the first clinicians to encounter such injuries in association with facial trauma are the oral and maxillofacial surgeons. Therefore, they perform the first line of ophthalmic examination and treatment in such cases. To lessen the severity of long-term effects and for legal reasons, a prompt diagnosis of a potentially serious ocular injury is crucial. It is important to give primary consideration to the treatment of eye problems. The visual results may be further harmed if the fractures are fixed before the ocular injuries are treated. A complete understanding of the ophthalmic complications associated with facial fractures is of prime importance as only dealing with the fractures at the cost of vision will itself be a treatment failure for oral and maxillofacial surgeons [1,15].

This prospective research set out to examine the links between ophthalmic complications and the distribution of maxillofacial fractures in India's northern regions, particularly the western Uttar Pradesh region.

## Materials and methods

Method of data collection

Throughout 2019-2021, 62 patients with maxillofacial fractures and ocular sequelae were included in a prospective study at the institution's department of oral and maxillofacial surgery approved by Teerthanker Mahaveer Dental College and Research Centre (ethical clearance number: TMDCRC/IEC/19-20/OMFS1). The inclusion and exclusion criteria are tabulated in Table 1.

**Table 1:** Inclusion and exclusion criteria

Inclusion criteria
Patients were selected irrespective of gender, age, religion, and socioeconomic status.
Patients with facial fractures causing ophthalmic injuries.
Patients who were willing to participate in the study.
Exclusion criteria
Pre-existing ophthalmic diseases.
Already treated fracture patients.
Old fractures, malunion of fractures.
Isolated mandible fractures.

Sequence of patient care

Maxillofacial surgeons conducted ocular examinations in all of these instances as part of their initial diagnostic workups. In the event of serious issues, ophthalmologists conducted a thorough evaluation. Under the direction of oral and maxillofacial surgeons and ophthalmologists, a chart review and data collection were performed to examine the relationship between maxillofacial trauma and ophthalmic problems.

Outcome measures

All patients were assessed to establish the occurrence of ophthalmic complications in relation to age, trauma mechanism, type of maxillofacial fracture, and prevalence of maxillofacial fractures in relation to trauma mechanism.

The correlation between ophthalmic complications and associated maxillofacial trauma was categorized into mild ophthalmic complications, moderate ophthalmic complications, and severe ophthalmic complications. Mild complications included periorbital edema, eyelid laceration, periorbital ecchymosis, and subconjunctival hemorrhage. Moderate complications included enophthalmos, proptosis, restriction of eye motility, diplopia, telecanthus, dystopia, mild vision loss, and dilated pupil. Severe complications included chemosis, hyphema, perforated lens, corneal abrasion, detached retina, retrobulbar hemorrhage, traumatic optic neuropathy (TON), and blindness.

Statistical analysis

Statistical Package for the Social Sciences (SPSS) version 19.0 (IBM Corp., Armonk, NY) was used for statistical analysis of the data collected for this investigation. Descriptive statistics included both frequency and percentages. The current investigation used a predetermined significance threshold of 5%. Chi-square analysis was used to compare the two separate groups.

## Results

Based on the gender distribution, 96.8% of the subjects were males and 3.2% were females. The age range of the patients was recorded as 11-52 years, with a mean age of 28.8 ± 7.87 years (Table 2).

**Table 2:** Age distribution of the subjects

	Range	Minimum	Maximum	Mean	Std. deviation
Age	41.00	11.00	52.00	28.80	7.87

The most common trauma mechanism was RTA in 43 patients (69.35%), followed by self-fall in 13 patients (20.96%) and assault in six patients (9.67%), as listed in Table 3.

**Table 3:** Prevalence of different types of injuries

Trauma mechanism	N	Percentage
Road traffic accident	43	69.35%
Assault	6	9.67%
Fall	13	20.96%

The type of maxillofacial fracture associated with ocular injuries, as illustrated in Table 4, mainly involved ZMC fracture (79%), mandibular fracture (48.4%), NOE fracture (22.6%), and isolated nasal fracture (16.1%). This was followed up by frontal bone, orbital floor, lateral orbital wall fracture (19.4%), medial orbital wall fracture (11.3%), Le Fort III fracture (9.7%), Le Fort II (4.8%), and Le Fort I fracture (3.2%). Conventional radiographs and computed tomography evaluations were done to confirm the diagnosis.

**Table 4:** Prevalence of various types of fractures among study subjects

Type of fracture	N	Percentage
Frontal bone	12	19.4%
Orbital floor	12	19.4%
Lateral orbital wall	12	19.4%
Medial orbital wall	7	11.3%
Nasal bone	10	16.1%
Naso-orbito-ethmoid	14	22.6%
Zygomaticomaxillary complex	49	79%
Le Fort I	2	3.2%
Le Fort II	3	4.8%
Le Fort III	6	9.7%
Mandible	30	48.4%

Prevalence rates of maxillofacial fracture with the trauma mechanism were statistically significant (p = 0.001), showing the highest number of patients with ZMC fracture in all categories of trauma mechanism, which was recorded as 34 (79.1%) in RTA, nine (69.2%) in falls, and six (100%) in assaults. The least prevalence rates were commonly recorded with Le Fort fractures as depicted in Table 5.

**Table 5:** Prevalence of maxillofacial fractures in relation to trauma mechanism

Facial bone involved	Road traffic accident (n = 43)	Assault (n = 6)	Fall (n = 13)
Frontal bone	9	1	2
	20.9%	16.7%	15.4%
Orbital floor	10	1	1
	23.3%	7.7%	16.7%
Lateral orbital wall	8	1	3
	18.6%	16.7%	23.1%
Medial orbital wall	3	2	2
	7.0%	33.3%	15.4%
Nasal bone	7	1	2
	16.3%	16.7%	15.4%
Naso-orbito-ethmoid	11	1	02
	25.6%	16.7%	15.4%
Zygomaticomaxillary complex	34	6	9
	79.1%	100.0%	69.2%
Le Fort I	1	0	1
	2.3%	0	7.7%
Le Fort II	1	0	2
	2.3%	0%	15.4%
Le Fort III	5	0	1
	11.6%	0%	7.7%
Mandible	20	2	8
	46.5%	33.3%	61.5%

The ophthalmic complications were divided into the mild, moderate, and severe groups. The majority of the findings were in the mild group, which included subconjunctival hemorrhage (90.32%), periorbital ecchymosis (62.90%), periorbital edema (29.37%), and eyelid lacerations (11.29%), as shown in Table 6.

**Table 6:** Prevalence of mild ophthalmic complications

Mild disorder	N	Percentage
Periorbital edema	18	29.03%
Eyelid laceration	7	11.29%
Ecchymosis	39	62.90%
Subconjunctival hemorrhage	56	90.32%

Subconjunctival hemorrhage was reported in all the cases of frontal bone (100%), orbital floor (100%), and Le Fort II fractures (100%). Ecchymosis was reported in all the fractures of the medial orbital wall, 90% of the isolated nasal bone fracture, and 83.30% of the frontal bone, orbital floor, and Le Fort III fractures. The highest prevalence of the periorbital edema was reported in the nasal bone fracture (100%), while none of the cases of the Le Fort II fracture reported periorbital edema. The highest prevalence of eyelid laceration was reported for frontal bone fracture (50%). The incidence of mild ophthalmic disorders in relation to various types of mid-facial fractures showed statistically significant results for all categories (p = 0.001), as depicted in Table 7.

**Table 7:** Incidence of mild ophthalmic complications in relation to maxillofacial trauma ZMC: zygomaticomaxillary complex fracture; NOE: naso-orbito-ethmoid fracture.

Mild disorder	Frontal bone (n = 12)	Orbital floor (n = 12)	Lateral orbital wall (n = 12)	Medial orbital wall (n = 7)	Nasal bone (n = 10)	NOE (n = 14)	ZMC (n = 49)	Le Fort I (n = 2)	Le Fort II (n = 3)	Le Fort III (n = 6)	Mandible (n = 30)
Periorbital edema	3	3	6	5	10	10	16	1	0	3	9
Eyelid laceration	6	0	1	0	2	1	7	0	0	0	1
Ecchymosis	10	10	7	7	9	11	33	1	2	5	19
Subconjunctival hemorrhage	12	12	10	6	9	13	47	1	3	5	27

Moderate ophthalmic complications included restrictions of ocular movements (9.68%), telecanthus (6.45%), proptosis (4.84%), dystopia (4.84%), enophthalmos (3.23%), diplopia (3.23%), and mild vision loss (1.61%), as shown in Table 8.

**Table 8:** Prevalence of moderate ophthalmic complications

Moderate disorder	N	Percentage
Enophthalmos	2	3.23%
Proptosis	3	4.84%
Restriction of ocular muscles	6	9.68%
Diplopia	2	3.23%
Telecanthus	4	6.45%
Dystopia	3	4.84%
Mild vision loss	1	1.61%
Dilated pupil	3	4.84%

The restriction of the ocular muscles was reported as 66.66% in Le fort III fractures. Telecanthus was reported in 57.14% of the medial orbital wall fracture and 40% of the nasal bone fractures. About 33.33% of the Le Fort III fractures reported proptosis. Dystopia was reported only in the orbital floor (16.66%), frontal bone (8.3%), and ZMC (6.1%). The highest prevalence of enophthalmos was reported in Le Fort I fracture (50%), followed by orbital floor fracture (16.66%). Diplopia was seen only in the fractures of the orbital floor (16.66%) and ZMC fractures (4.1%). The highest prevalence of the fixed dilated pupil was seen in the Le Fort III fractures (16.66%), nasal bone fracture (10%) orbital floor fractures, frontal bone, lateral orbital wall (8.3%), and NOE fractures (7.14%). Table 9 describes the incidence of moderate ophthalmic disorders in relation to various types of mid-facial fractures showing statistically significant results (p = 0.001).

**Table 9:** Incidence of moderate ophthalmic complications in relation to maxillofacial trauma ZMC: zygomaticomaxillary complex fracture; NOE: naso-orbito-ethmoid fracture.

Moderate disorder	Frontal bone (n = 12)	Orbital floor (n = 12)	Lateral orbital wall (n = 12)	Medial orbital wall (n = 7)	Nasal bone (n = 10)	NOE (n = 14)	ZMC (n = 49)	Le Fort I (n = 2)	Le Fort II (n = 3)	Le Fort III (n = 6)	Mandible (n = 30)
Enophthalmos	0	2	1	0	0	1	2	1	0	0	1
Proptosis	1	2	0	0	0	1	2	0	1	1	1
Restriction of ocular muscles	0	2	0	1	1	1	4	0	2	1	4
Diplopia	0	2	0	0	0	0	2	0	0	0	1
Telecanthus	0	0	1	4	4	3	3	0	0	1	3
Dystopia	1	2	0	0	0	0	3	0	0	0	1
Mild vision loss	0	1	0	0	0	1	1	0	0	1	0
Fixed dilated pupil	1	1	1	0	1	1	3	0	0	1	0

Severe ophthalmic findings included hyphemia, chemosis, corneal abrasions, and retrobulbar hematoma, each including 3.23%, and TON leading to blindness in 1.61% (Table 10).

**Table 10:** Prevalence of severe ophthalmic complications

Severe disorder	N	Percentage
Chemosis	2	3.23%
Hyphema	2	3.23%
Perforated lens	0	0%
Corneal abrasion	2	3.23%
Detached retina	0	0%
Retrobulbar hematoma	2	3.23%
Traumatic optic neuropathy	1	1.61%
Blindness	1	1.61%
Others	0	0%

Hyphemia was reported in 16.66% of cases of the lateral orbital wall and Le Fort III fractures. Chemosis was seen in the nasal bone (20%), lateral orbital wall (16.67%), Le Fort III (16.67%), and medial orbital wall fractures. Corneal abrasion and retrobulbar hematoma were seen in 33.33% of the Le Fort III fractures. TON was seen in only one patient with Le Fort III fracture. Complete blindness was reported in one patient with ZMC fracture, Le Fort III fracture, and NOE fracture. Table 11 describes the severe ophthalmic disorders in relation to mid-facial trauma with statistically significant results (p = 0.001).

**Table 11:** Incidence of severe ophthalmic complications in relation to maxillofacial trauma ZMC: zygomaticomaxillary complex fracture; NOE: naso-orbito-ethmoid fracture; TON: traumatic optic neuropathy.

Severe disorder	Frontal bone (n = 12)	Orbital floor (n = 12)	Lateral orbital wall (n = 12)	Medial orbital wall (n = 7)	Nasal bone (n = 10)	NOE (n = 14)	ZMC (n = 49)	Le Fort I (n = 2)	Le Fort II (n = 3)	Le Fort III (n = 6)	Mandible (n = 30)
Chemosis	1	0	2	1	2	0	2	0	0	1	0
Hyphema	0	2	0	0	0	1	2	0	0	1	0
Corneal abrasion	1	0	0	1	1	2	2	0	0	2	0
Retrobulbar hematoma	0	1	0	0	0	1	1	0	0	2	0
TON	0	0	0	0	0	0	0	0	0	1	0
Blindness	0	1	0	0	0	1	1	0	0	1	0

## Discussion

Because of their more exposed location, the face, orbit, and eyes are more likely to sustain an injury during an accident. The direction and impact of the traumatic force that caused the fracture, as well as the degree of damage that was received by the facial tissues, can all contribute to the formation of a unique fracture pattern in the facial region. Ophthalmic injuries happen with varying frequency and variety despite the eye's protection by the orbital bones. The ophthalmic problems that may arise range from minor to severe problems such as blindness from globe rupture, retinal detachment, and acute optic neuropathy [3,9].

The majority of maxillofacial fractures (about 70%) in our research were reported to have been caused by RTAs. Our findings are consistent with those of Agarwal et al., who found an RTA incidence of 64.4%, and those of Mittal et al., who found an RTA incidence of 71.3% [8,16]. The second leading etiology for facial trauma in our study was fall (20.9%), which shows accordance with the other study conducted by Agarwal et al. (25.1%), which was conducted in Uttar Pradesh and does not correlate with the other Indian studies such as Septa et al., which presented assaults as the second leading etiology for facial trauma [9,16].

Maxillofacial injuries are more prevalent in men than women as shown in this study with 97% of males being affected by the injuries. Cultural and socioeconomic factors have a key role in shaping the gender gap in injury rates, as shown by Septa et al. [9]. Maxillofacial injuries are more common in males than in women, according to data from a study by Al Ahmed et al. [17]. Similar to how men are more likely to conduct outside labor due to cultural norms in Uttar Pradesh, this may be a contributing factor to why there are fewer women than men who meet RTAs [18].

Patients in the middle age group (mean age: 28.8 years) had the highest incidence of maxillofacial fractures in the current study, which is consistent with the majority of previous studies that report the mean age as falling somewhere between the second and fourth decades when people are most active [19]. The analysis showed that ZMC fractures made up the bulk of the cases followed by fractures to the orbital walls, NOE, and Le Fort. People who had pan facial fractures were more likely to have issues related to their eyes. Major problems were often the result of ocular damage that occurred with a fracture of the orbital wall or rim. Orbital wall and rim fractures, as well as midface and NOE fractures, increased the risk of problems for patients.

In this research, ophthalmic injuries were found to be relatively mild in 98.38% of cases, moderate in 29.03%, and severe in 12.90% of cases. Similar percentages of moderate and severe problems were seen in the research by Al-Qurainy et al. [11]. Similar findings were seen in a study done by Mittal et al., where 29.03% had mild problems and 9.6% experienced severe ones [8].

The subconjunctival hemorrhage was the most prevalent ocular problem we saw. Patients presenting with facial injuries most often exhibit periorbital edema and ecchymosis, both of which are included in the category of mild sequelae. Similarities were seen between the results of our investigation and the work of Septa et al. [9].

Evaluation of ocular movements was done to rule out any mechanical entrapment (confirmed by computed tomography scan and forced duction test) and/or paresis of ocular muscles leading to restricted eyeball movement [20]. In the present study, patients with severe edema of the ocular region leading to restricted and painful ocular movements were also recorded. In such cases, observation and drug therapy were advised till the edema subsided.

Diplopia is a visual impairment that may cause the sufferer extreme distress. If caused by paresis, it gradually improves with time. Muscle entrapment should be checked out as a possible cause of persistent diplopia. Ocular floor fractures and medial wall fractures often cause entrapment [1]. Restrictions in the extraocular movement were seen in 9.68% of patients overall, with 3.2% exhibiting diplopia specifically. Of patients with midfacial fractures, 19.8% had diplopia in the research of Al-Qurainy et al., but Kamath et al. reported no incidences of diplopia [11,21].

Midface fractures and/or orbital wall fractures may cause lateral, inward, or outward displacement of the globe, leading to telecanthus, enophthalmos, or exophthalmos, respectively. In the event of orbital wall fractures or inferior/lateral displacement of the zygoma with the orbital floor, an increase in the infraorbital volume will occur, leading to enophthalmos. Displacement of medial canthal attachments, most often caused by destruction or displacement of medial orbital walls, is the hallmark of traumatic telecanthus [18,22,23]. Our study findings showed enophthalmos (3.23%) and telecanthus (6.45%) in patients concurrent with NOE fractures and Le Fort III fractures. A study conducted by Rajkumar et al. showed similar results where 5% of cases were reported with telecanthus [4].

**Figure 1:**
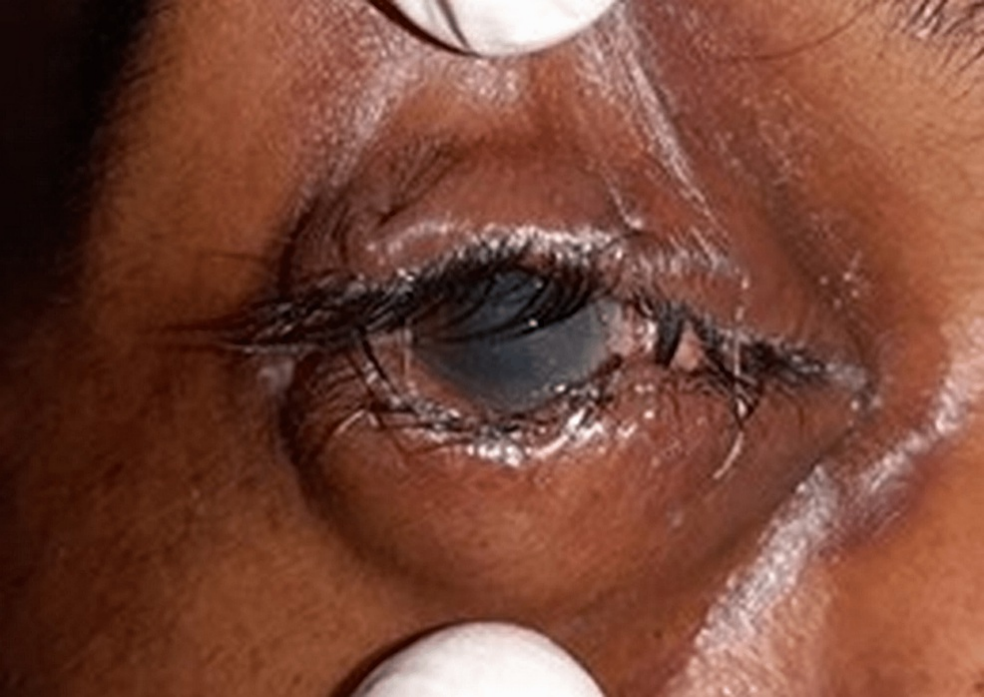
Examination of the eye showing hazy cornea and ill-defined iris details

The incidence of intraocular injuries such as traumatic hyphema (caused by the ripping of blood vessels at the iris root) was somewhat lower than in previous research [24,25]. One patient was discovered to have a corneal laceration and cloudy cornea (Figure 1).

**Figure 2:**
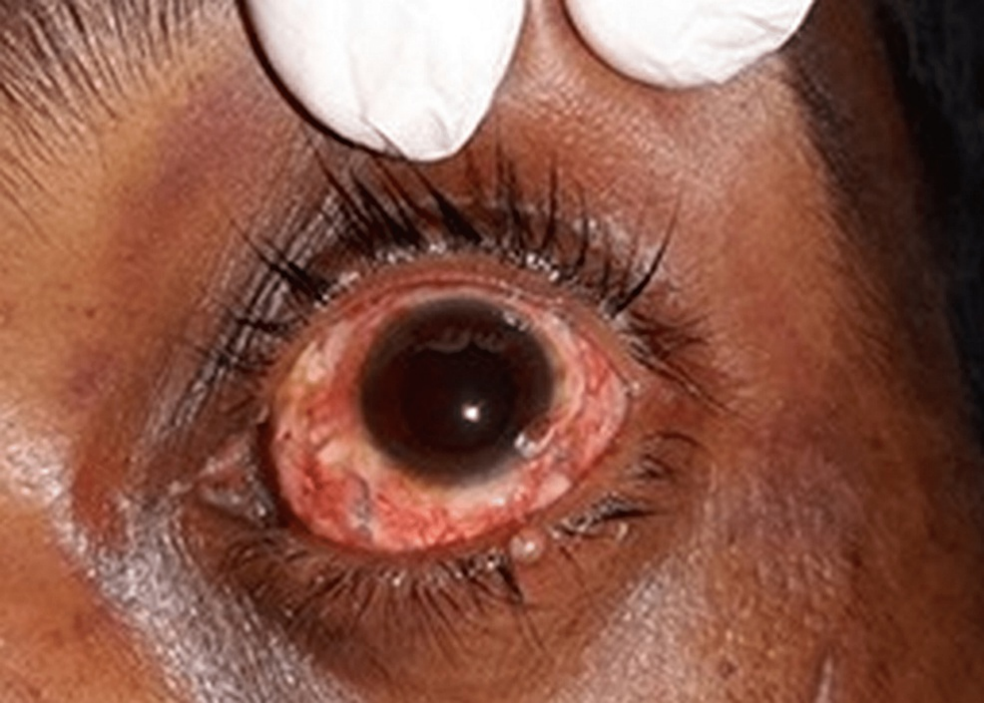
Examination of the eye showing abnormal scleral thickenings and dilated fixed pupils

Subluxated lenses, retinal/vitreous hemorrhages, and scleral tears were not seen in our investigation. Ophthalmologists diagnosed a globe rupture in one of our research participants with aberrant scleral thickenings and dilated fixed pupils on fundoscopic inspection (Figure 2).

As a result of its rigid bony framework and firm anterior soft tissue cover, the orbit is particularly vulnerable to the development of compartment syndrome in the event of an injury within the limits of the extraocular muscle (intraconal space), which tears the vessels of the posterior ciliary artery. The high pressure in the orbit may affect the vascular supply to the optic nerve, choroids, and retina, leading to vision loss following craniofacial trauma [18]. One participant in our trial had a retrobulbar hemorrhage. The patient reported on day seven post-incident that his visual loss has been becoming worse since day three. The patient was found to have retrobulbar hemorrhage and a ruptured globe, both of which might have caused the patient's blindness. Case reports of blindness after maxillofacial trauma have varied widely among research studies. Injuries to the eyeball, retrobulbar hemorrhage that compresses the optic nerve, or traumatic optic neuropathy might all be potential causes [18,23]. Ansari and Eidlitz-Markus et al. elucidated that immediate, permanent blindness mostly results from compression of displaced bone whereas delayed progressive vision loss results from nerve compression due to underlying hemorrhage or interstitial edema [26,27].

The findings of moderate and severe ocular complications were relatively low as compared to minor complications in our study. This is the universal finding of all the studies given in the literature. This can be attributed to the impact of injury as the majority of cases reported in our study were of low to moderate complications, leading to comparatively less complex ocular findings.

We propose as a part of the study's limitations more sample inclusions and observations in the future with the involvement of several centers to gain a better knowledge of facial fractures that cause ophthalmic injuries.

## Conclusions

We conclude by saying that mid-facial fractures, especially the ZMC fractures, showed the majority of the ophthalmic complications, followed by orbit, NOE, and Le Fort II and III fractures. Severe complications leading to blindness were very less, yet were demotivating and devastating for the patient. So, every patient with mid-facial trauma should undergo a thorough ophthalmic evaluation and when in need or suspected of major morbidity, should undergo a complete examination by an ophthalmologist for proper timely treatment. Because of the urgency of treating certain ocular injuries, such as optic nerve compression, this is crucial information, even if it does not change the kind of fracture reduction and fixation that is used.
